# Whole-body MRI-based long-term evaluation of pediatric NF1 patients without initial tumor burden with evidence of newly developed peripheral nerve sheath tumors

**DOI:** 10.1186/s13023-024-03420-6

**Published:** 2024-11-04

**Authors:** Marie-Lena Schmalhofer, Said Farschtschi, Lan Kluwe, Victor Felix Mautner, Gerhard Adam, Lennart Well, Inka Ristow

**Affiliations:** 1 Department of Diagnostic and Interventional Radiology and Nuclear Medicine, University Medical Center Hamburg-Eppendorf, Martinistraße 52, 20246 Hamburg, Germany; 2 Department of Neurology, University Medical Center Hamburg-Eppendorf, Martinistraße 52, 20246 Hamburg, Germany

**Keywords:** Peripheral nerve sheath tumor, NF1 in children, Plexiform neurofibromas, Magnetic resonance imaging, MRI-based long-term evaluation

## Abstract

**Background:**

Patients with neurofibromatosis type 1 (NF1) can develop plexiform neurofibromas (PN). Large tumor burden is a predictor for the development of malignant peripheral nerve sheath tumors. Whole-body magnetic resonance imaging (WB-MRI) is the recommended imaging method for the evaluation of PN. WB-MRI is recommended for NF1 patients at transition from adolescence to adulthood. In the absence of internal PN further follow-up WB-MRI is not considered necessary. PN are often detected in early childhood, leading to the assumption that they may be congenital lesions. It remains unclear whether this invariably applies to all patients or whether patients who initially displayed no tumors can still develop PN over time. Therefore, we retrospectively reviewed WB-MRI scans of pediatric patients with NF1 without initial tumor burden and compared these with long-term follow-up scans for presence of newly developed PN.

**Methods:**

We retrospectively reviewed WB-MRI scans of 17 NF1-children (twelve male; median age at initial scan: 9 [IQR 6.1–11.9] years) who initially displayed no PN. MRI scans with a follow-up interval of at least 6 years (median follow-up interval: 9 [IQR 5.6–12.4] years) were reviewed in consensus by two radiologists regarding the development of new PN over time.

**Results:**

New PN were identified in two out of 17 children without initial tumor burden in follow-up examinations. One of these two patients developed two larger distinct PN of 4.5 cm on the right upper arm and of 2.5 cm on the left thoracic wall between the age of ten and twelve. The second child developed multiple smaller PN along the major peripheral nerves between the age of eleven and 16. In addition, 15 of the children without initial tumor burden did not develop any distinct tumors for a period of at least 6 years.

**Conclusion:**

Our results indicate that PN can be newly detected in pediatric patients over time, even if no PN were detected on initial MRI scans. Therefore, it seems reasonable to perform at least a second MRI in pediatric NF1 patients at transition to adulthood, even if they did not display any tumor burden on initial MRI, and when the MRI was performed significantly under the age of 18. With this approach, tumors that may have developed between scans can be detected and patients at risk for complications can be identified.

## Introduction

Neurofibromatosis type 1 (NF1) is a neurogenetic disorder with a highly variable natural history and phenotypic expression [1, 2]. It is considered one of the most common hereditary neurocutaneous diseases with a birth incidence of one in 2500–3000 newborns [3, 4]. NF1 is caused by pathogenic variants in the *NF1*-tumor suppressor gene coding for the protein neurofibromin [5–7]. Approximately 50% of patients with NF1 Arising from peripheral nerves is redundant of the skin, subcutis, and the deeper soft tissue, so-called plexiform neurofibromas (PN) [5, 8]. These benign peripheral nerve sheath tumors can involve multiple nerve fascicles or branches of the major nerves, can cause significant morbidity due to disfigurement, pain, local compression, or loss of function of nerves [9, 10], and contribute to a reduced quality of life and mental disorders [11, 12].

Previous studies have investigated the incidence and growth rate of PN in children with NF1 and have shown, among other findings, that PN are often detected in early childhood, leading to the assumption that they may be congenital lesions [13–16].

Distinct nodular lesions (DNL) describe PN with a characteristic appearance on MRI. These lesions are well-circumscribed, encapsulated-appearing, measure ≥ 3 cm in size, and can be present within or outside of PN. Previous studies showed that most of these distinct nodular lesions had atypical features in the histologic evaluation, thus representing premalignant precursors [17–21]. Rapid increase in tumor size, development of new neurological deficits, and severe pain are indicative of a malignant transformation [8, 22, 23]. Since these highly malignant tumors respond poorly to radio- and chemotherapy, early detection and complete surgical resection is the only curative therapy [24]. Vice versa, determining the malignant potential is of great importance for the extent of the surgical approach and consecutively for the patient´s outcome. However, early detection of malignant transformation remains a clinical challenge, as there are no specific symptoms, and malignant tumors are usually deep-seated and not always accessible by physical examination alone.

Whole-body magnetic resonance imaging (WB-MRI) is the imaging method of choice for screening, early diagnosis, and long-term monitoring of PN in patients with NF1 [25]. According to the tumor surveillance guidelines from the ERN GENTURIS (European Reference Network for GENetic TUmor RIsk Syndromes), annual examinations including history taking and a clinical examination to detect complications of NF1 are recommended for children up to 10 years. A WB-MRI is recommended at least for all NF1 patients at the transition from adolescence to adulthood to evaluate internal tumor burden as a predictor for malignant transformation [26]. In the absence of internal PN usually no further monitoring using WB-MRI is recommended [26].

However, it remains unclear whether pediatric patients who display no tumors on initial MRI scans still can develop PN over time. As we know, PN may grow rapidly in children and adolescents, suggesting that young patients require closer monitoring by WB-MRI and clinical examination [15]. However, there are only a few studies on the MRI-based evaluation of the development of peripheral nerve sheath tumors in NF1 in children. Given the paucity of literature discussing this relation, we aimed to retrospectively review WB-MRI scans of pediatric patients with NF1 without initial tumor burden and to compare these with long-term follow-up scans for the presence of newly developed PN.

## Materials and methods

### Study population

This study protocol has been approved by the local Institutional Review Board (Ärztekammer Hamburg; 2023-300412-WF; app. date 23.11.2023) for retrospective analysis of the available data which complied with the Declaration of Helsinki.

Inclusion criteria were children with a diagnosis of NF1 according to the National Institutes of Health Diagnostic Criteria [27] or/and available results of genetic testing for NF1 as well as the availability of high-resolution MRI examinations at 1.5 or 3 Tesla with axial and coronal T2-weighted fat-suppressed MRI sequences. No distinct nodular lesions were allowed to be present on the initial WB-MRI, and patients had been followed up by MRI for at least 6 years. The MRI studies were acquired between 2005 and 2022.

### General characteristics

The inclusion criteria were fulfilled by 17 children (twelve male) from a total cohort of 350 NF1 patients. The median age at initial MRI was 9 years [IQR 6.1–11.9; range 1–13]. The median follow-up interval was 9 years [IQR 5.6–12.4; range 6–15] and the median number of follow-up examinations was 4 [IQR 5.4–2.6; range 2–7]. Detailed information on patient characteristics is provided in Table 1.

**Table 1:** Patient characteristics

Case-ID	sex	Age at initial MRI (years)	Age at last MRI (years)	FU period (years)	Number of FU	Genetic variant	Stigmata	PN, distinct nodular lesions
1	m	13	19	6	2	Frameshift; > 99% function loss	Scoliosis, small NOF intraosseous tibia, small NF < 1 cm	–
2	m	7	16	9	3	Not studied	Small NF < 2 cm	–
3	f	12	26	14	3	Stop gain; > 99% function loss	NF < 2.5 cm	–
4	m	8	14	6	3	Missense (noonan); > 99% function loss	Dural ectasia, small NF < 1 cm	–
5	m	6	14	8	4	No pathogenic variants found	Dural ectasia, NF < 2.5 cm	–
6	m	12	22	10	4	No pathogenic variants found	Dural ectasia, NF < 3 cm, small NOF intraosseous tibia	–
7	f	8	20	12	4	Stop gain; > 99% function loss	Dural ectasia, NF < 2.5 cm	–
8	m	9	18	9	4	Frameshift; > 99% function loss	Small NF < 1 cm, small NOF intraosseous femoral	–
9	f	9	24	15	5	Frameshift; > 99% function loss	Small NF < 1 cm	–
10	m	11	17	6	3	Not studied	Dural ectasia, NF < 3 cm	–
11	m	8	15	7	3	Not studied	Small NF < 1.5 cm	–
12	m	1	12	11	6	Not studied	Small NF < 2 cm	–
13	m	12	18	6	3	Frameshift; > 99% function loss	Small NF < 1.5 cm	–
**14**	**m**	**8**	**24**	**16**	**7**	**Stop gain; > 99% function loss**	**Dural ectasia**	**Right upper arm (4.5 cm), left thoracic wall (2.5 cm), multiple smaller NF along all larger peripheral nerves**
**15**	**f**	**7**	**22**	**15**	**6**	**Missense, splicing; > 95% function loss**	–	**Diffuse neurofibromas along all larger peripheral nerves**
16	f	10	16	6	4	Stop gain; > 99% function loss	Small NF < 1 cm	–
17	f	10	18	8	3	Frameshift; > 99% function loss	Scoliosis, dural ectasia, small NF < 1.5 cm	–

MRI, Magnetic resonance imaging; FU, Follow-up; NF, Neurofibromas; PN, Plexiform neurofibromas; f, Female; m, Male; NOF, Non-ossifying neurofibroma; lines with a bold font correspond to the patients who developed new tumors during the observation period

### MR imaging

MR imaging was performed at 1.5 or 3 Tesla (Siemens Healthineers, Erlangen, Germany). The imaging protocol included a localizer, coronal, and axial T2-weighted fat-suppressed sequences (including short tau inversion recovery [STIR] or Turbo-Inversion Recovery-Magnitude [TIRM]). Distinct nodular lesions or plexiform neurofibromas were detected based on their characteristic appearance as signal-intense lesions on T2-weighted sequences. MRI studies were reviewed in consensus by two radiologists ( LW and IR) with 10 and 5 years of experience in imaging of NF1-associated tumors regarding the development of new PN over time.

### Genetics

Genetic data were available for 13 out of 17 children. DNA from blood samples was screened for pathogenic variants of the NF1 gene by means of either Sanger Sequencing or targeted sequencing using a custom panel. For the targeted sequencing, a library containing these amplicons was prepared for each DNA from blood of a patient and was sequenced on an iSeq100. The resulting data were evaluated by an integrated “amplicon analysis module” which gave variants that deviated from the reference sequence. The genomic position is given according to the GRch37 genome and the variants are given in the HGVS nomenclature [28]. Clinical significance of the variants was classified using the ENIGMA criteria [29].

### Statistics

The evaluation was based on descriptive observations and therefore no statistical analysis was performed.

## Results

### Development and growth prediction of PN

The majority of the pediatric NF1 patients (15 of 17 children) without initial tumor burden did not develop any distinct tumors on follow-up MRI scans for at least 6 years. However, most of the 15 children (87%) developed morphological characteristics such as skeletal malformations (e.g., scoliosis), non-ossifying neurofibroma (NOF)-like bone lesions, or multiple small cutaneous as well as subcutaneous neurofibromas (Table 1).

**Fig. 1:**
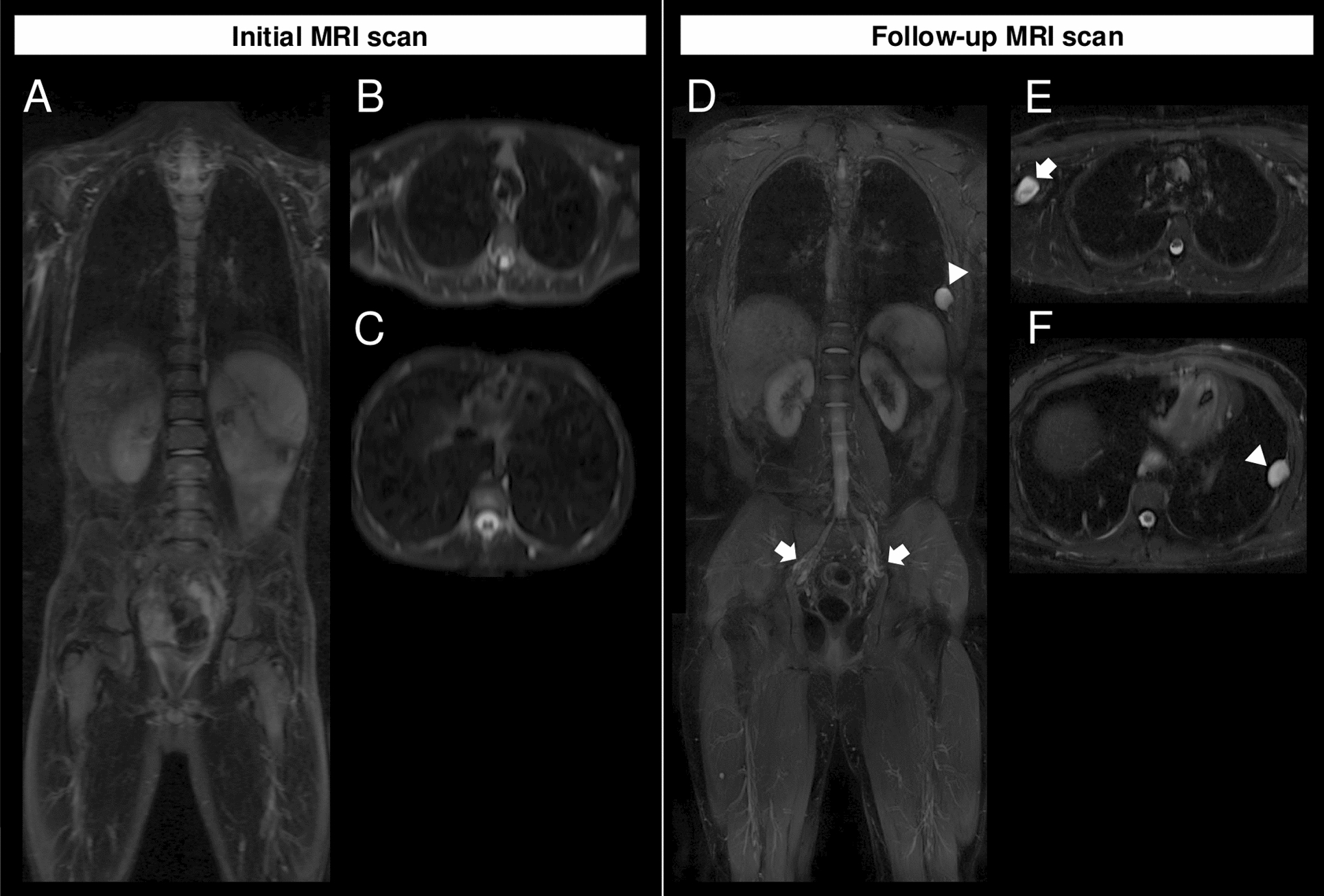
Patient example 1 with newly detected PN during follow-up MRI. **A** Coronal and **B**, **C** axial T2-weighted, fat-saturated MRI sequences of the upper body acquired at 1.5T in a 10-year-old male, who displayed no peripheral nerve sheath tumors at the initial scan in 2007. (**D** Follow-up examination in 2021, coronal T2-weighted, fat-saturated MRI sequence acquired at 3T at the age of 24, showing a newly developed larger distinct nodular lesion in the left thoracic wall (arrowhead) as well as multiple smaller neurofibromas (arrows). **E**, **F** Axial T2-weighted, fat-saturated MRI of the follow-up examination in 2021 with newly developed larger distinct nodular lesions in the left thoracic wall (arrowhead) and in the right upper arm (arrow)

**Fig. 2:**
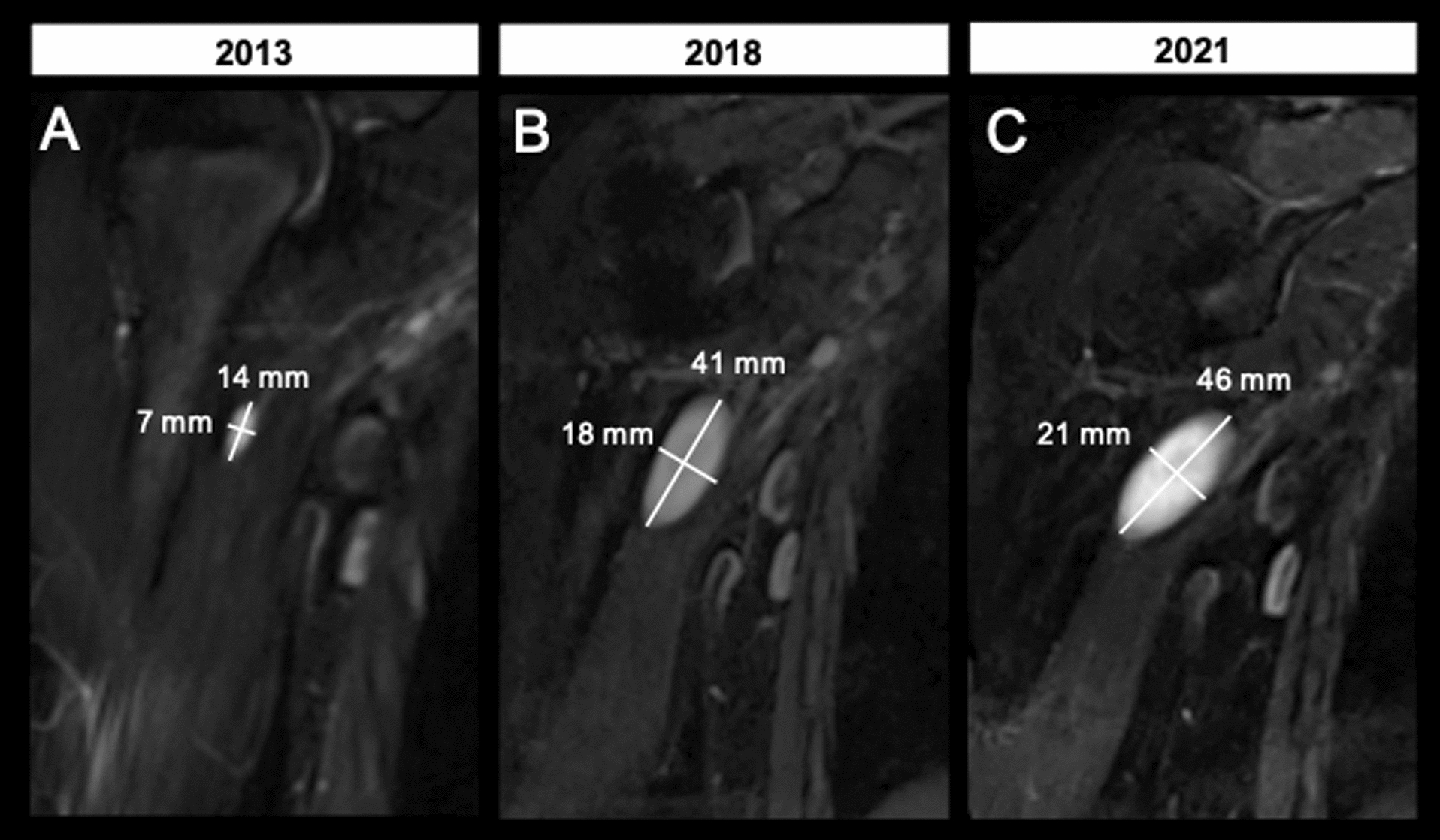
Long-term tumor growth characteristics of the distinct nodular lesion in the right upper arm of patient example 1. **A** Coronal T2-weighted, fat-saturated MRI of the right upper arm of a 16-year-old male, acquired at 1.5T in 2013 showed a newly developed lesion measuring 14 mm × 7 mm. **B** Follow-up examination in 2018, acquired at 3T at the age of 21, revealed an increase in size to 41 mm × 18 mm (growth of 192.9% in the long axis). **C** Follow-up examination in 2021 acquired at 3T at the age of 24 showed an increase in size of the lesion to 46 mm × 21 mm (percentage growth of 12.2% in the long axis). Assuming continuous growth pattern, the median tumor growth rate per year was 12.5%

**Fig. 3:**
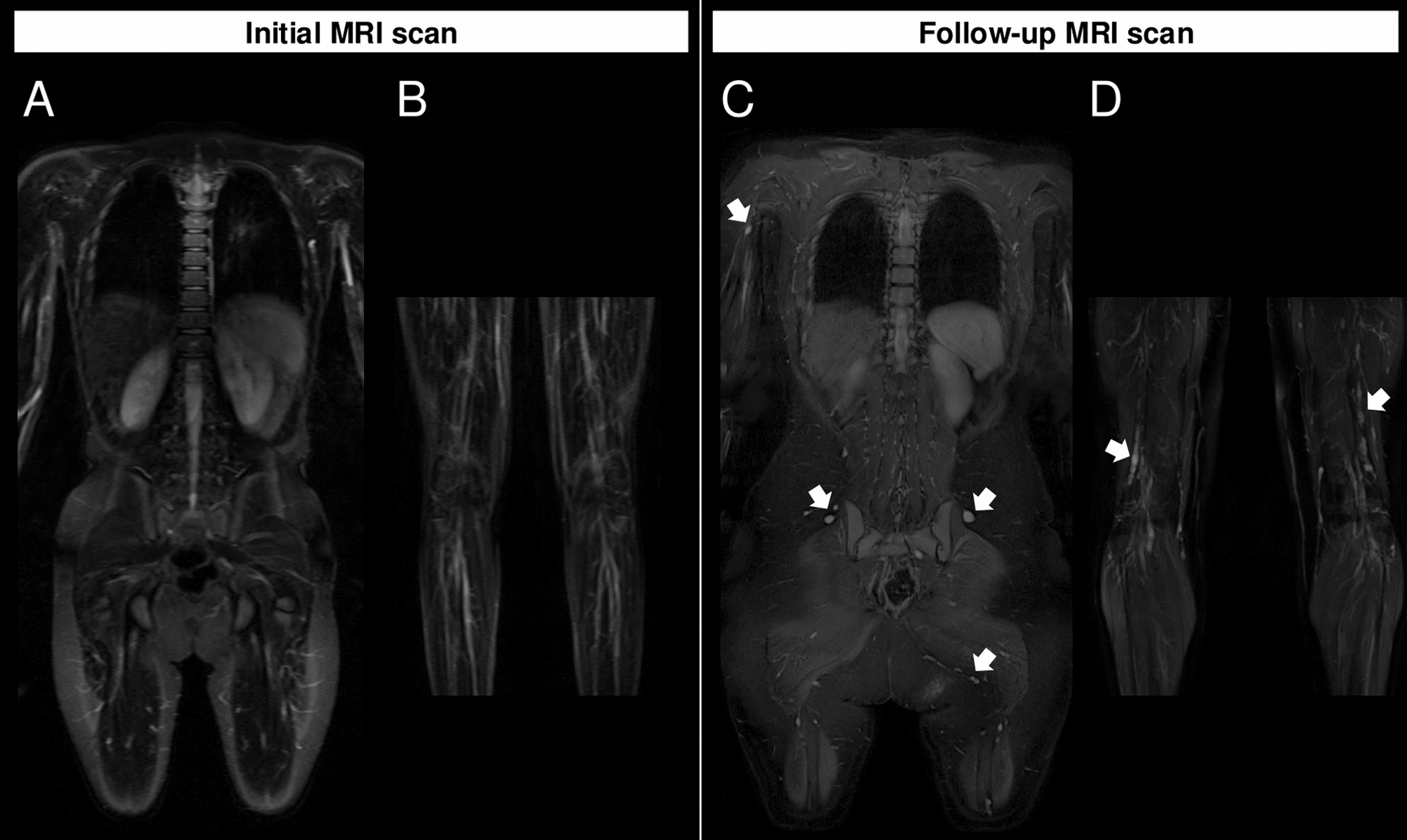
Patient example 2 with newly detected PN during follow-up MRI. **A**, **B** Coronal T2-weighted, fat-saturated MRI sequences of the body and the upper limbs acquired at 1.5T in a 9-year-old female, who displayed no peripheral nerve sheath tumors at the initial scan in 2007. **C**, **D** Follow-up examination in 2020 at the age of 22 shows multiple newly developed smaller neurofibromas along all larger peripheral nerves (arrows) on the coronal T2-weighted, fat-saturated MRI sequence of the body and the upper limbs at 3T

In two out of 17 children without initial tumor burden, new PN were identified on follow-up MRI scans. One of these two patients developed two larger PN of 4.5 cm on the right upper arm and of 2.5 cm on the left thoracic wall between the age of ten and twelve as well as multiple smaller PN along the major peripheral nerves (Fig. 1). The growth pattern of the larger distinct nodular lesion in the right upper arm of patient example 1 over the period 2013–2021 is shown in Fig. 2, illustrating tumor growth during adolescence. The second child developed multiple PN along the major peripheral nerves between the age of eleven and 16 (Fig. 3). Note the differences in tissue contrast, especially the fat-suppression related to the different scanners in 1.5T and 3T. No child showed a malignant transformation in the observed period.

## Discussion

The current study retrospectively examined WB-MRI scans of pediatric patients with NF1 without initial tumor burden and compared them with long-term follow-up scans (follow-up period ≥ 6 years) regarding the presence of newly developed PN and distinct nodular lesions. New PN were identified in two out of 17 children without initial tumor burden on follow-up examinations between the age of ten and twelve and eleven and 16, respectively. Thus, our results indicate that PN can be newly detected in pediatric patients over time, even if no PN were present on initial MRI scans.

WB-MRI is the current reference standard for the detection of NF1-associated tumors and plays an important role in patient management by providing important information about the localization and extent of PN as well as signs of potential malignant transformation due to its excellent soft tissue contrast [30]. According to the ERN GENTURIS tumor surveillance guidelines annual assessments to detect complications of NF1 are recommended for children up to 10 years, and a WB-MRI is recommended for all NF1 patients during transition from adolescence to adulthood to evaluate internal tumor burden [26]. In the absence of internal PN, no further monitoring using WB-MRI is usually recommended [26].

Previous studies have investigated the incidence and growth rate of PN in children with NF1. For example, Kang et al. reported that about one-third of NF1 patients at the age of three have PN, and progressive growth was observed in about 30% of patients [13]. Among other things, Waggoner et al. evaluated the age of tumor detection and the localization of PN in 68 NF1 patients and reported that PN were detected at birth in six of 68 patients [14]. Furthermore, twelve of 72 PN were diagnosed in the first year of life (including six of these noted at birth) and 32 of 72 PN were diagnosed before the fifth year of life [14]. Additional studies have shown that the growth rate of PN in NF1 patients is inversely correlated with age, with younger patients exhibiting the highest growth rates. The median percentage of change in PN volume per year was 21.1% in children younger than 8.3 years [15]. The young age at detection supports the hypothesis, that these tumors are congenital lesions. Here we report new detection of PN in follow-up studies in pediatric patients with NF1 around the age of twelve and between the ages of eleven and 16.

One possible reason for the new detection of PN in follow-up studies could be that the tumors can develop over the course of a lifetime. To our knowledge, there are hardly any studies investigating the new development of PN, so further studies are required. Another reason for the detection of new PN in two children could be that smaller neurofibromas were not detected in the preliminary examinations, for example, due to the poorer spatial in plane resolution and/or the large slice thickness of six to eight millimeters at initial MRI. Therefore, these imaging findings cannot be used to generally contradict the perception of PN being congenital tumors, although they question the hypothesis. Overall, this emphasizes the importance of WB-MRI in NF1 patients to identify PN growth and assess potential malignant transformation at an early stage. WB-MRI should also be considered in (adult) patients even when no significant tumor load was present in childhood. This is strikingly important as NF1 patients may develop also other tumors which can be less symptomatic than their sporadic counterparts, such as pheochromocytomas, GIST-tumors, or high-grade gliomas [26, 31]. However, one of the major concerns in pediatric imaging is the need for sedation, which is why the indication should be discussed thoroughly in a multidisciplinary team [32].

Limitations of the current study include the rather small sample size as well as the only medium-long follow-up interval of more than 6 years due to the retrospective analysis. Further research, including long-term studies with longitudinal WB-MRI scans and clinical evaluation of children and adolescents after completion of longitudinal growth is needed to gain further insight into the development of PN in NF1 patients. Another limitation is the large slice thickness of six to eight millimeters as well as the difference in MRI resolution at 1.5T compared to 3T. A higher field strength MRI (3T) has a better signal-to-noise ratio than an MRI at 1.5T and thus creates significant differences in tissue contrast between the scanners, especially the fat-suppression [33, 34]. As mentioned above, it could therefore be possible that smaller neurofibromas were not detected in the preliminary scans due to the poorer resolution which were then detected as PN in the follow-up scan. Additionally, spinal abnormalities can only be assessed to a limited extent as no sagittal sequences were acquired.

## Conclusion

Our results may indicate that PN can be newly detected in pediatric NF1 patients over time, even if no PN were detectable on initial MRI scans. Therefore, it seems reasonable, and in accordance with the ERN guidelines, to perform at least a second MRI in pediatric NF1 patients at transition to adulthood, even if they did not display any tumor burden in initial MRI, and when the MRI was performed significantly under the age of 18. With this approach, tumors that may have developed between the scans can be detected and patients at risk for complications can be identified. One possible reason for the new detection of PN in follow-up studies could be that the tumors can develop over time, but further research, including long-term studies, is needed in this regard.

## Data Availability

Due to the protection of individual patient data, the datasets generated during and/or analyzed during the current study are available from the corresponding author upon reasonable request.
